# Endocannabinoid System Unlocks the Puzzle of Autism Treatment *via* Microglia

**DOI:** 10.3389/fpsyt.2021.734837

**Published:** 2021-10-22

**Authors:** Tangfeng Su, Yu Yan, Qiang Li, Jiacai Ye, Lei Pei

**Affiliations:** ^1^Department of Pediatrics, Tongji Hospital, Tongji Medical College, Huazhong University of Science and Technology, Wuhan, China; ^2^Department of Neurology, People's Hospital of Dongxihu District, Wuhan, China; ^3^Exchange, Development and Service Center for Science and Technology Talents, The Ministry of Science and Technology, Beijing, China; ^4^Department of Radiation Oncology, Affiliated Cancer Hospital and Institute of Guangzhou Medical University, Guangzhou, China; ^5^Collaborative Innovation Center for Brain Science, The Institute for Brain Research, Huazhong University of Science and Technology, Wuhan, China; ^6^Department of Neurobiology, School of Basic Medicine, Tongji Medical College, Huazhong University of Science and Technology, Wuhan, China; ^7^Department of Anesthesiology, Washington University in Saint Louis School of Medicine, Saint Louis, MO, United States

**Keywords:** endocannabinoid system, microglia, autism spectrum disorder, immune, neurodevelopmental disorders

## Abstract

Autism spectrum disorder (ASD) is a serious neurodevelopmental disorder and characterized by early childhood-onset impairments in social interaction and communication, restricted and repetitive patterns of behavior or interests. So far there is no effective treatment for ASD, and the pathogenesis of ASD remains unclear. Genetic and epigenetic factors have been considered to be the main cause of ASD. It is known that endocannabinoid and its receptors are widely distributed in the central nervous system, and provide a positive and irreversible change toward a more physiological neurodevelopment. Recently, the endocannabinoid system (ECS) has been found to participate in the regulation of social reward behavior, which has attracted considerable attention from neuroscientists and neurologists. Both animal models and clinical studies have shown that the ECS is a potential target for the treatment of autism, but the mechanism is still unknown. In the brain, microglia express a complete ECS signaling system. Studies also have shown that modulating ECS signaling can regulate the functions of microglia. By comprehensively reviewing previous studies and combining with our recent work, this review addresses the effects of targeting ECS on microglia, and how this can contribute to maintain the positivity of the central nervous system, and thus improve the symptoms of autism. This will provide insights for revealing the mechanism and developing new treatment strategies for autism.

## Introduction

Autism spectrum disorder (ASD) is a group of heterogeneous neurodevelopmental disorders characterized by language and social dysfunctions and repetitive stereotyped behavior. In addition to the core diagnostic features, ASD also has a variety of comorbid symptoms, including aggression, hyperactivity, epilepsy, anxiety, sleep disorders, gastrointestinal disorders and immune dysfunction. The global incidence rate is about 1% and a male to female ratio of about 3 to 4:1 (1).

**Figure 1:**
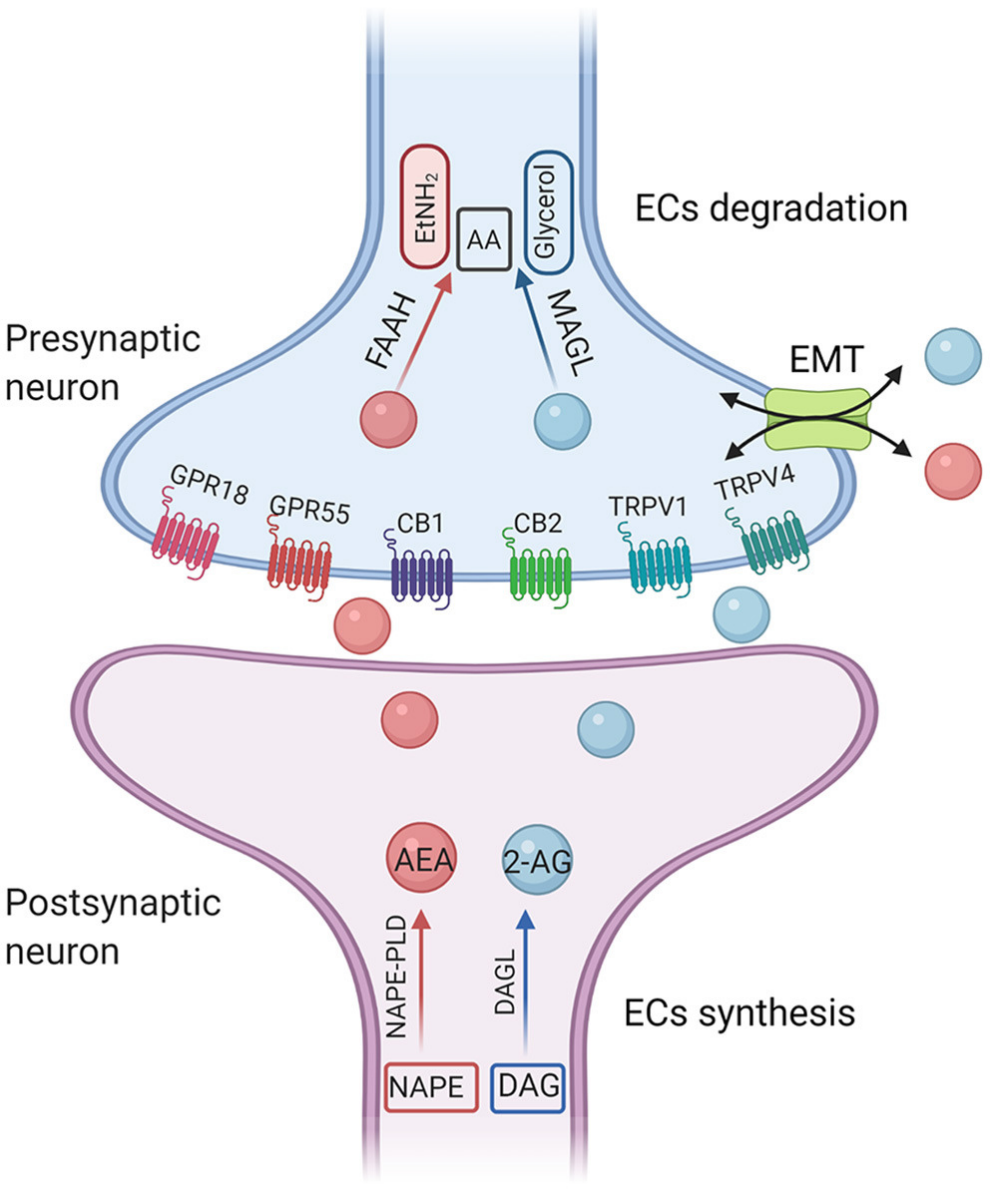
A simplified illustration of endocannabinoid synthesis and degradation pathway. The diacylglycerol lipases (DAGLα and DAGLβ) hydrolyze DAG to generate 2-AG. DAGL-α is expressed on postsynaptic neurons within various brain regions. In contrast, DAGL-β is most highly expressed on microglia. 2-AG is preferentially degraded by monoglyceride lipase (MAGL). The main synthetase of AEA is N-acyl-phosphatidylethanolamine-phospholipase D (NAPE-PLD). The degradation enzyme of AEA is fatty amide hydrolase (FAAH). AEA, anandamide; 2-AG, 2-arachidonoyl-glycerol; CB1, cannabinoid receptor 1; CB2, cannabinoid receptor 2; TRPV1, transient receptor potential cation channel subfamily V member 1; GPRs, orphan G-protein-coupled receptors; AA, arachidonic acid; EtNH2, ethanolamine; EMT, extraneuronal monoamine transporter.

Currently, there is no pharmaceutical compound available to alleviate the core symptoms of ASD. Risperidone and aripiprazole have been approved by the US Food and Drug Administration (FDA) to treat irritability and behavioral disorders in patients with ASD. Long-term use of these drugs may cause some side effects, including sedation and weight gain (2). At present, the most widely used treatment of ASD is behavior intervention, but it is usually time-consuming and unaffordable. In recent years, oxytocin (OT) has attracted wide attention because it plays an important role in regulating the function of social communication in the central nervous system (CNS), and has gradually become a potential treatment for autism. For example, recent evidence supports entry of intranasal oxytocin into the brain through the olfactory and trigeminal neural pathways (3), as well as the nasal blood stream (4). Intranasal administration of the OT can enter the CNS through the blood-brain barrier, but the OT molecule is very likely to enter the vein, that is, the peripheral nervous system, which can cause uterine contraction in women. Therefore, almost all nasal spray OT experimental studies on the treatment of ASD were conducted on males (5). There may be gender differences in the effect of OT on ASD, so it is questionable to extend the results to all ASD patients. The Endocannabinoid system (ECS) plays a putative role in the control of neural processes of controlling social anxiety and social reward, and has become a new target for autism intervention in the last decade (6). The ECS includes the two most widely studied endogenous lipid mediators [N-arachidonoylethanolamide (anandamide; AEA), and 2-arachidonoylglycerol (2-AG)], cannabinoid type 1 and type 2 receptors (CB1 and CB2), and endogenous lipid mediator synthesis and catabolism enzymes (Figure 1). AEA and 2-AG were identified in 1992 and 1995, respectively. CB1 and CB2 were cloned in 1990 and 1993, respectively. The amino acid sequence homology of CB1 across species is 97–99%, which is mainly found in the brain, especially in neuronal cells, while CB2 are mainly found in immune cells. The two receptors have 44% homology (7). Lines of evidence demonstrated that targeting different components of ECS may provide therapeutic strategies for the treatment of ASD (8), but the mechanism is unclear.

It is reported that dysregulation of the immune system is a possible mechanism related to ASD pathogenesis and severity (9). Furthermore, microglia are the main producers of endocannabinoids under neuroinflammatory conditions (10). Here, we will review studies concerning the involvement of the ECS in the immuno-pathological mechanism and therapeutic strategies of ASD by summarizing preclinical and clinical studies to shed light on ECS and microglia as a potential target for the treatment of autism.

## Materials and Methods

A Medline (PUBMED) search using the terms “microglia AND autism,” or “microglia AND autism spectrum disorder,” or “microglia, endocannabinoid AND autism,” or “microglia, cannabidiol AND autism,” or “microglia, endocannabinoid AND autism spectrum disorder,” or “microglia, cannabidiol AND autism spectrum disorder” was performed. A total of 339 articles with a publication date up to April 1st, 2021 were included. Articles related to clinical trials, preclinical studies, and animal studies were selected for this review.

## The Endocannabinoid System: A Potential Target for the Treatment of Autism

### 2-AG

2-AG is the most abundant endogenous cannabinoid in the brain and its concentration is about 200 times that of AEA (11). The diacylglycerol lipases (DAGLα and DAGLβ) hydrolyze DAG to generate 2-AG. DAGL-α is expressed on postsynaptic neurons within various brain regions (12). In contrast, DAGL-β is most highly expressed on microglia (13). 2-AG is preferentially degraded by monoglyceride lipase (MAGL). Fragile X mental retardation 1 (*FMR1*) gene is one of the most common single-gene mutations associated with autism. It was reported that 2-AG-mediated decreased long term depression (LTD) in nucleus accumbens (NAc) and medial prefrontal cortex (mPFC) in *FMR1* knockout mice. Administration of JZL184, a pharmacological inhibitor of MAGL, enhanced 2-AG signaling, corrected 2-AG-mediated LTD, and improved hyperactivity and anxiety-like responses in *FMR1* knockout mice (14). In addition, a single mutation of *Shank3* is sufficient to induce the typical autism syndrome (15). In terms of the neural circuits in the treatment of ASD, studies have shown photogenetic activation of the glutamatergic circuit in basolateral amygdala-nucleus accumbens (BLA-NAc) reduced social activity and increased social avoidance in *Shank3B*^−/−^ mice. Systemic or local injection of 2-AG hydrolase inhibitor JZL184 into the brain area of NAc restored the social deficit of *Shank3B*^−/−^ mice. At the same time, electrophysiological recordings in mouse brain slices *in vitro* showed that JZL184 corrected the abnormal excitatory and inhibitory neural transmission of NAc in *Shank3B*^−/−^ mice, and reduced the feedforward inhibition of BLA on NAc neurons (16).

### AEA

Compared with 2-AG, studies have shown that AEA is more specific in mediating social reward response (17). The main synthetase of AEA is the N-acyl-phosphatidylethanolamine-phospholipase D (NAPE-PLD), and the degradation enzyme of AEA is the fatty amide hydrolase (FAAH). Pharmacological inhibition of FAAH enhances AEA levels and reverses the behavioral deficits among different autistic animal models. *FMR1* knockout mice displayed increased anxiety-related behaviors during social interaction. It was reported that modulation of AEA signaling can ameliorate some aspects of the behavioral phenotype in *FMR1* knockout mice. Qin et al. used acute injection of FAAH inhibitor URB597 to improve aversion memory and relieve anxiety behavior in *FMR1* knockout mice, but it did not affect social behavior (18). However, Wei et al. showed that blocking FAAH by acute administration of URB597 completely reversed the social impairment in *FMR1* knockout mice (19), suggesting that increased AEA may play a prosocial role in ASD animal models. The different time points of pre-administration of URB597 [30 min (18) and 3 h (19), respectively] or the different mouse line [C57Bl/6J (18) and FVB/NJ (19), respectively] may give an explanation for the discrepancy reported in the two studies.

Both of the two FAAH inhibitors, PF3845 and URB597, can reverse the abnormal behaviors of male offspring of the valproic acid (VPA) model in SD rats (20, 21). It is noteworthy that prenatal exposure to VPA produced gender-specific differences in the rat model: the behavioral impairment of male rats exposed to VPA was more serious than that of female rats. Increasing AEA signaling by inhibiting AEA-degrading enzyme FAAH improved the behavioral impairment of both male and female offspring of the ASD model. Compared with male offspring VPA rats, female offspring were less vulnerable in social communication, emotional response and cognitive performance, while selective deficiencies in social games and stereotyped behavior were observed (22). This gender dichotomy is similar to the clinical phenotype of ASD in humans. The phenotypic differences of ASD between males and females may be partly related to abnormally activated microglia during early development, which leads to impaired microglia-mediated synaptic pruning, especially in males (23). A recent study found that URB597 could restore ECS-related synaptic plasticity by enhancing the AMPA receptors transportation in mPFC of the offspring of the VPA model, thus alleviating ASD-related behavioral symptoms (24).

In addition, autism has been associated with atypical sleep patterns and circadian rhythms (25). Polymorphisms in ASMT (involved in melatonin synthesis), paralleled by reduced levels of circulating melatonin, have been reported in autism (26). Interestingly, the EC system also plays a role in regulating circadian rhythms (27), thus making it a putative target to examine using animal models of autism-related phenotypes.

### Cannabinoid Receptors

CB1 is widely distributed both in the central nervous system (CNS) and peripheral nervous system, participates in synaptogenesis, learning and memory, and regulates a variety of physiological activities by regulating neurohormone levels and signaling transduction. CB2 is mainly expressed in the immune system, such as the spleen margin and thymus of the peripheral immune organs. During inflammation or other pathological injuries, the expression of CB2 in the CNS, especially in glial cells, is significantly up-regulated (10). CB1 antagonist rimonabant can alleviate cognitive impairment, epileptic sensitivity, and hyperalgesia in *FMR1* knockout mice. At the biochemical level, CB1 blockade corrected the excessive activation of mTOR signaling and restored the morphology of dendritic spines in the hippocampus of *FMR1* knockout mice. It is worth noting that the treatment with CB2 antagonist AM630 can also reduce the sensitivity of anxiety-like behavior and audiogenic epilepsy, indicating that both CB1 and CB2 are involved in the behavior of Fragile X syndrome (FXS) (28). Another study also found that low doses of rimonabant and CB1 neutral antagonist NESS0327 prevented cognitive impairment in *FMR1* knockout mice. The therapeutic effect of rimonabant on cognitive impairment is related to the functional recovery of LTD induced by metabotropic glutamate (mGlu) in the hippocampus of *FMR1* knockout mice (29). In addition, the behavioral phenotype of inbred BTBR mice is consistent with the core symptoms of ASD. Multiple changes of neuroanatomy and histology of BTBR mice are similar to those of ASD, which make BTBR mice widely used in ASD study. Wei et al. reversed social impairment in BTBR mice by using FAAH inhibitor URB597 to increase AEA levels, which was not attributed to reduced anxiety. The reason is that FAAH inhibition did not change the performance of BTBR mice in elevated mazes. In addition, the effect of inhibition of FAAH on the social pathway in BTBR mice strictly depends on the enhancement of AEA in CB1 signaling pathway, since the CB1 antagonist AM251 can prevent the recovery of social ability mediated by URB597, which is not related to the alteration of 2-AG content (19).

Recently, imbalances in dopamine levels have been implicated in behavioral disorders such as schizophrenia, autism and depression. Neurochemical evidence shows that activation of CB1R expressed on Ventral tegmental area (VTA) GABA neurons inhibits GABAergic transmission and in turn stimulates dopaminergic neurotransmission in the NAc (30). Since the dopamine signaling abnormalities have been reported in both autistic patients (31) and ASD animal models (32), elucidating the relationship between ECS and DA in ASD will help to provide a better understanding of the etiopathogenesis of ASD along with new therapeutic strategies (33).

### Clinical Case Studies

The changes of ECS in peripheral blood can be used as a new non-invasive diagnostic index for several neuroinflammatory diseases (34). Compared with healthy children, the expression of AEA synthase NAPE-PLD mRNA in peripheral monocytes of children with ASD was downregulated (35). Using improved liquid chromatography-tandem mass spectrometry, it was found that the AEA concentration in peripheral blood of children with ASD was lower than that of healthy children (36). The levels of endogenous cannabinoids AEA, OEA, and PEA in serum samples were lower than those in age-and sex-matched normal control groups, but there was no significant difference in 2-AG between the two groups (37). Cannabidiol (CBD) is a non-psychoactive component in the cannabis plant, which inhibits FAAH by activating peroxisome proliferator-activated receptor (PPAR) and transient receptor potential channels of vanilloid type-1 (TPRV1), and finally compensates for the decrease of AEA, OEA, and PEA levels in children with ASD. The Clinical Trials (Registration number: NCT02956226) conducted by an Israeli research team in 2017 for the first time investigated the effectiveness of cannabis drugs against behavioral problems in children with ASD. Nighty-three children with ASD and severe behavioral problems (age: 11.8 ± 3.5 years, 83% were boys) tolerated the use of drugs rich in CBD (the ratio of CBD to THC was 20:1). Sixty-three percentage of the children reported considerable amelioration in behavior problems associated with ASD. Improvement was also found in anxiety and communication problems in 39 and 47% of the children, respectively (38). Another clinical study reported that CBD treatment reduced comorbidities in multiple domains, including self-harm behavior, anger attacks, hyperactivity symptoms, sleep problems, and anxiety states (39). The average duration of CBD treatment for ASD children in the study was 66 days, with mild side effects, including mild drowsiness and appetite changes.

## Microglia and Autism

Genetics and early environmental factors play a key role in the etiology of ASD. Although disruptions of immune system, abnormal neurotransmission, mitochondrial deficiency, oxidative stress, aberrations of cell signaling transduction, and epigenetic alterations have been proven to be associated with ASD, the pathophysiological mechanisms of ASD remain poorly understood, and no single etiology is identified. Microglia have detrimental impacts on several key etiological factors of autism, such as the synaptic plasticity, neural circuitry, brain immune system, stem cell development, and the crosstalk between genetic and environmental factors. Postmortem studies found immune-related genes were upregulated, while the synaptic function-related genes in the brain of ASD patients were downregulated compared with the control group (40). Both genetic and environmental factors could contribute to abnormal synaptic pruning and immune responses mediated by microglia either *in utero* or during the early postnatal period, which may underlie the pathogenesis of ASD. An association between maternal immune activation (MIA) and ASD in offspring was reported in human epidemiological studies (41) and in rodent MIA models (42). Disruptions in the composition of the gut microbiota during early developmental stages due to perinatal events increase an individual's predisposition to autistic behavioral patterns (43).

### Microglia and Neuroinflammation in Autism

A large number of studies have shown that immune abnormality is one of the most important factors in the occurrence of ASD (44). Microglia have long been thought to originate from peripheral macrophages that enter the brain after birth. However, it is now known that microglia develop from red marrow progenitor cells in the yolk sac of early embryos and migrate into the brain (45). Microglia are the innate immune effector cells in the CNS and are the first line of defense against CNS infection and injury. Microglia can be activated by any type of pathological events or changes in brain homeostasis and release cytokines, chemokines and growth factors to maintain normal brain function. Abnormal immune signaling and microglial function is consistently demonstrated in postmortem brain tissues from autistic individuals as well as in autism mouse models. Transcriptomic analyses of postmortem cortex tissue of ASD patients revealed that genes associated with activated microglia were upregulated with exhibited exaggerated M2 activation states (46). Early lipopolysaccharide (LPS) exposure rats showed impairment in communication and cognition, which is associated with M2-like microglia activation and enhanced neurogenesis in both the subgranular zone (SGZ) and the subventricular zone (SVZ) (47). Of note, a recent systematic review comprising 1,007 autism patients from 14 genetic or neuroimaging studies using PET/MRI collectively indicates that microglia mediated neuroinflammation plays a causative role in the pathogenesis of ASD. Those prominent manifestations of microglia in autistic patients include increased cell number or cell density, morphological alterations, and phenotypic shifts (48). Despite this, microglial activation may have diverse meanings in different patients, playing pathogenic roles among “dysimmune” patients (49) and leading downstream consequence in “neural disconnection” ASD patients (50).

### Microglia and Synaptic Pruning in Autism

Microglia play an important role in maintaining a healthy brain by engulfing improper and less active synapses. Thus, microglial dysfunction is hypothesized to be involved in the pathogenesis of ASD, and possibly through the mechanism of attenuated or excessive synaptic pruning. For example, microglia give priority to phagocytosis of weaker or less active synapses, promoting the development of functional neural circuits with stronger or more active synapses (51). Proper synaptic pruning is essential for the development of functional neural circuits. Defects in synaptic pruning disrupt the excitatory and inhibitory balance of synapses. For example, inhibition of microglia autophagy can lead to an increase in synaptic density and a decrease in social ability in mice (52). Chemokine CX3C receptor 1 (CX3CR1) is specifically expressed in microglia and mediates neuron-microglial interactions (53). In *CX3CR1* knockout mice, the expression of IL-1β was robustly increased and the synaptic long-term potentiation (LTP) was significantly decreased (54). In addition, functional magnetic resonance imaging (fMRI) analysis showed that the functional connection between hippocampus and prefrontal cortex was impaired. It is noteworthy that those *CX3CR1* knockout mice showed decreased social interaction and increased repetitive behavior similar to ASD (55).

A number of studies have shown that there are abnormalities in the morphological characteristics of microglia in the brain of postmortem ASD patients. Tetreault et al. found that the density of microglia in two cortical regions (frontal lobe and visual cortex) of ASD patients was significantly higher than that in the normal brain (56). Tang et al. reported that the dendritic density of layer V pyramidal neurons in Brodmann 21 area of the middle temporal gyrus, which is mainly involved in social interaction and communication, is increased in postmortem ASD specimens (57). The density of dendritic spines in the childhood ASD group (2–9 years old) was similar to that in an age-matched control group, while the dendritic spine density in the adolescent ASD group (13–19 years old) was significantly higher than that in age-matched healthy controls (58). Recently, large-scale sequencing studies and transcriptomic genome-wide analyses have identified two broad categories of genes, neuronal genes and transcriptional regulation genes, which contains more than 100 *de novo* mutations or variants that encode proteins for synaptic formation, transcriptional regulation and chromatin-remodeling in ASD (59). Therefore, excessive microglial activation could be the consequence of abnormal neurodevelopment leading to unstable and malfunctioning long-range synaptic connections.

Besides being a key player in the brain's immune system, microglia also play a crucial role in synaptic pruning. Recently, studies have clarified complement-mediated synaptic pruning by microglia in the visual thalamus (60). In the dorsal lateral geniculate nucleus (LGN), the classical complement cascade mediates microglia's functions (61). Both the complement protein C1q, and the downstream complement protein C3 are highly localized to immature synapses and are required for synaptic pruning in the retinogeniculate system. C1q expression was increased in the peripheral serum of children with ASD (62). Alternatively, ubiquitously increased complement proteins may cover the specific localization of complement proteins on less active synapses, which may promote excessive synaptic pruning by microglia. Recently, Thomas and colleagues proposed the atypical synaptic over-pruning hypothesis by a neurocomputational model that ASD is caused by the exaggeration of a normal system-wide phase of brain development, elimination of excess connectivity. Normal individual differences in the onset or rate of this phase interact with the pathological pruning process to create different trajectories of atypical development. Individual differences in other neurocomputational parameters and in environmental stimulation operate as risk or protective factors. The atypical pruning is assumed to impact more on long-range connectivity, impairing integrative functions, which leads to the unique behavioral profile of ASD (63).

### Microglia and Maternal Immune Activation in Autism

Environmental factors can also alter the functions of microglia, thereby affecting synaptic connections and development of the brain. Viral and bacterial infections during pregnancy are associated with an increased risk of autism in offspring (64). During neural tube closure, exposure to LPS induced maternal immune activation (MIA) in rodents seems to be associated with ASD-like behavior in offspring (65). Prenatal MIA and the subsequent increase in pro-inflammatory cytokines increase the risk of ASD and schizophrenia in the offspring (66).

Human epidemiological studies and MIA animal models have reported the association between MIA and offspring ASD. In MIA animal models, pregnant mice are immunologically activated by injection of polyinosine and polycytidine or LPS during embryonic development (E9-12). These MIA models will help to study the possible role of microglia in the pathogenesis of ASD, as offspring exhibit a range of ASD-like behavioral phenotypes, including impaired social interaction, changes in repetitive behavior, anxiety, and ultrasonic vocalization. LPS-exposed rodents at early postnatal days showed deficits in social interaction and induced a robust microglia activation, which is characterized by mixed microglial proinflammatory (M1) and anti-inflammatory (M2) phenotypes (47). A growing body of rodent studies have demonstrated that diverse inflammatory factors beyond infection, including stressors or environmental toxins, may similarly induce persistent effects on microglia, which in turn affect neurodevelopmental processes that lead to ASD-like phenotypes in offspring (67).

### Microglia and Gut-Brain Axis in Autism

The MIA-induced ASD mouse model not only showed abnormal behavior, but also had changes in intestinal microflora (68). The communication between microbes in the gastrointestinal tract and the brain regulates mood and behavior through a two-way brain-gut pathway. It is known that gastrointestinal dysfunction affects inflammatory response and brain function through nerve, hormone and immune signaling, but the exact biological mechanism remains to be elucidated. Compared with healthy people, patients with ASD are more likely to develop gastrointestinal symptoms, including constipation, diarrhea, or abdominal pain, as well as other intestinal diseases, such as inflammatory bowel disease (IBD) (69).

Sharon et al. found that normal intestinal microbial population is necessary for the development of microglial structure and function to mature. Compared with mice fed in a specific pathogen free (SPF) environment, axenic mice showed significant differences in the microbial mRNA expression profile, microglial density in multiple brain regions and process branching complexity and process length of microglia (70). SPF mice are free of a specific list of pathogens, such as disease-causing pathogens. However, axenic mice are free of all microorganisms, including those that are typically found in the gut. After virus infection, the microglia in axenic mice showed a decreased innate immune response, which indicated that the host microbial population supported the resistance of microglia to bacterial and viral stimulation. Compared with SPF mice, aseptic mice showed lower social ability and more repetitive self-grooming behavior (71). At the same time, aseptic mice had more immature and malformed microglia and reduced immune response to LPS or virus infection (72). It is worth noting that the microglial phenotype and social impairment in aseptic mice were rescued after intestinal reproduction using microflora from typically developed mice. As the microglia activation and the subsequent inflammation in the CNS may partly result from microbial disorders, it is important to analyze the integrity of mucosal barrier and the changes of intestinal microbial population in those disease models.

## Endocannabinoid System and Microglial Functions in Autism

**Figure 2:**
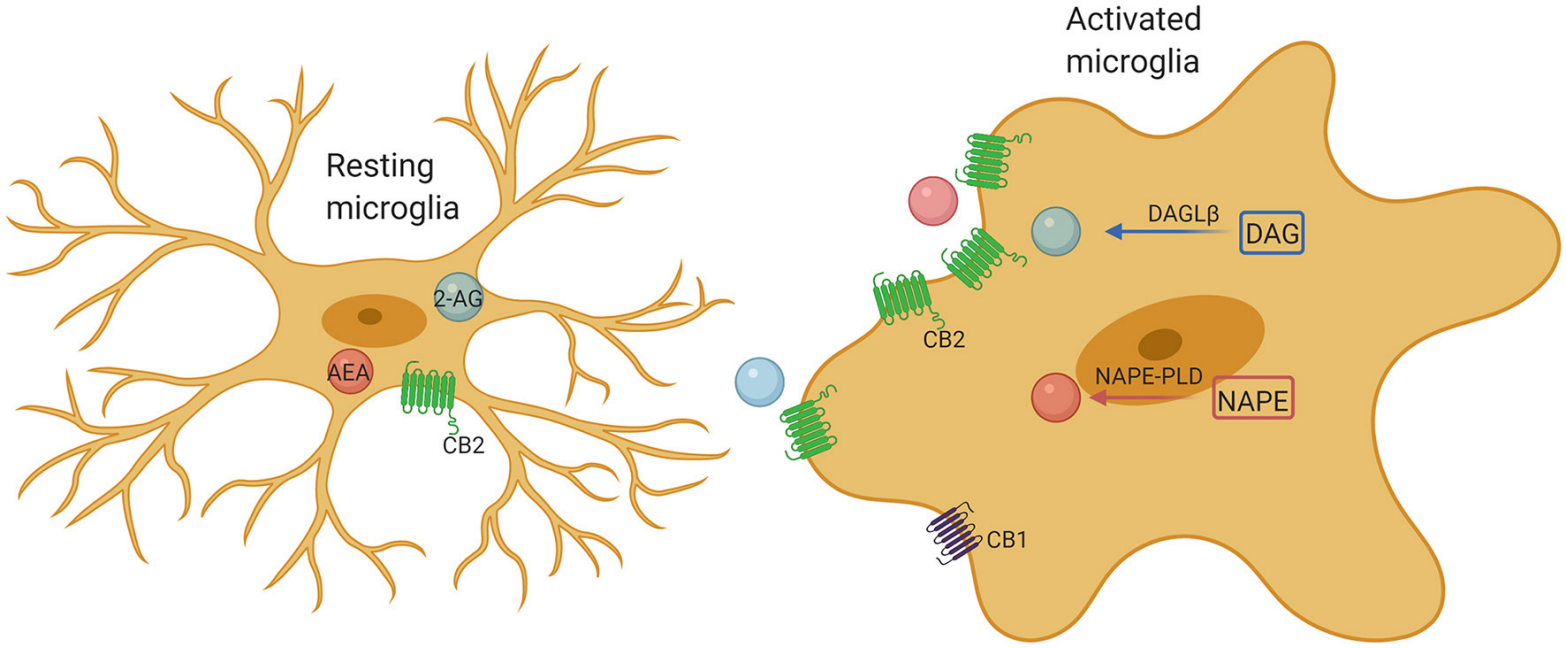
Microglia comprise a complete endocannabinoid signaling system. Microglia produce ~20-fold more endocannabinoids than neurons and other glial cells, and may be a major cellular source of endogenous cannabinoids under neuroinflammatory conditions. Upon activation, microglia markedly increase their endocannabinoids synthesis and upregulate their CB2 receptor expression, promoting protection of the microglia phenotype by enhancing their neuroprotective factor production and reducing their proinflammatory factor release.

It is known that CB1 is widely expressed in neurons, while CB2 is mainly expressed in immune cells and microglia. In the pathological state, the expression of CB2 in microglia significantly increased (10). The different activation states are considered to represent different functional states of microglia. Considerable studies have demonstrated that ECS may be a promising tool for changing the outcome of inflammation, especially by affecting the activity of microglia. Microglia contain a complete functional ECS signaling system, including synthesis and degradation components (Figure 2). The expression of cannabinoid receptors (mainly CB2) and the production of endogenous cannabinoids (eCB) are related to the activation characteristics of these cells. Previous study has shown that the ECS is closely associated with regulating polarization of microglia (73). Indeed, activation of CB2 receptors not only affects the migration, proliferation and releasing of proinflammatory cytokines of microglia, but also affects the phagocytosis and promotes the transformation of microglial cells to the anti-inflammatory M2 phenotype from M1 phenotype (10). Both CB1 and CB2 receptors are downregulated in M1 microglia, whereas the M2a and M2c microglia show phenotypic changes in the endocannabinoid machinery, such as M2a favors 2-AG synthesis and M2c favors AEA (10). CB1 is important for neuronal differentiation, normal axon migration and the establishment of neuronal connectivity. Interference in critical periods is considered to have adverse effects on brain development (74, 75). Overall, microglia in ASD are usually found more shifted toward the M2 state than M1 (76). This predominance of the M2 state would seem to point toward a compensatory role of microglial activation, not a pathogenic role, which would better explain the lack of efficacy of minocycline in ASD (77). Although stimulation of CB2 receptors promotes the shifting of microglia to the M2 state, it is still unclear whether overstimulating CB2 receptors will produce any further improvement. However, chronic CB2 activation has not been found to produce any tolerance, while CB1 activation is associated with withdrawal effects in the CNS (77). Understanding the effects of overstimulating CB2 receptors remains a topic of great interest. Hopefully, future research will address this issue.

Doenni et al. found that early inflammation caused by a single injection of LPS on postnatal day 14 reduced the social play and non-play behavior of adolescents in male and female rats. The social defect induced by LPS is related to the decreased binding of eCB with CB1, elevated AEA levels, and increased FAAH in the amygdala. Oral administration of FAAH inhibitor PF-04457845 (1 mg/kg) before social testing could normalize LPS-induced social behavior changes. A similar improvement was observed after direct injection of PF-04457845 into the basolateral amygdala, suggesting that changes in AEA signaling in this brain region play a key role in mediating LPS-induced social dysfunction (78).

Targeted activation of CB1 may bring some side effects to the CNS, such as depression, suicidal tendency, and other mental disorders (79). However, activation of CB2 receptor does not cause these central side effects. On the contrary, activation of CB2 can modulate inflammatory immune response and is becoming a potential intervention target for immune regulation, neuroinflammation and neurodegenerative diseases. Zamberletti et al. report that cannabidivarin (CBDV) treatment at two postnatal stages (postnatal day19–32 and postnatal day 34–58), respectively, bring beneficial therapeutic effect on the VPA autism rat model. Early preventive treatment relieves deficits in social novelty, impairment of short-term memory, and hyperactivity without affecting stereotyped behavior. Late rehabilitation therapy can improve social impairment, social novelty preferences, short-term memory deficit repetitive behavior and hyperactivity. CBDV treatment upregulated CB2 protein in the hippocampus, restored the expression of GFAP, CD11b, and TNF- α in the same brain region, promoted the activation of microglia, and further demonstrated its direct or indirect neuroprotective effect (80). Our previous work also found that CB2 activation reduced the release of proinflammatory cytokines such as IL-1β, IL-6, and TNF-α in the peripheral skin of rats with inflammatory pain (81). Based on those studies, ECS intervention may regulate neuroimmune inflammation through CB2 to alleviate ASD symptoms.

## Future Perspectives

**Figure 3:**
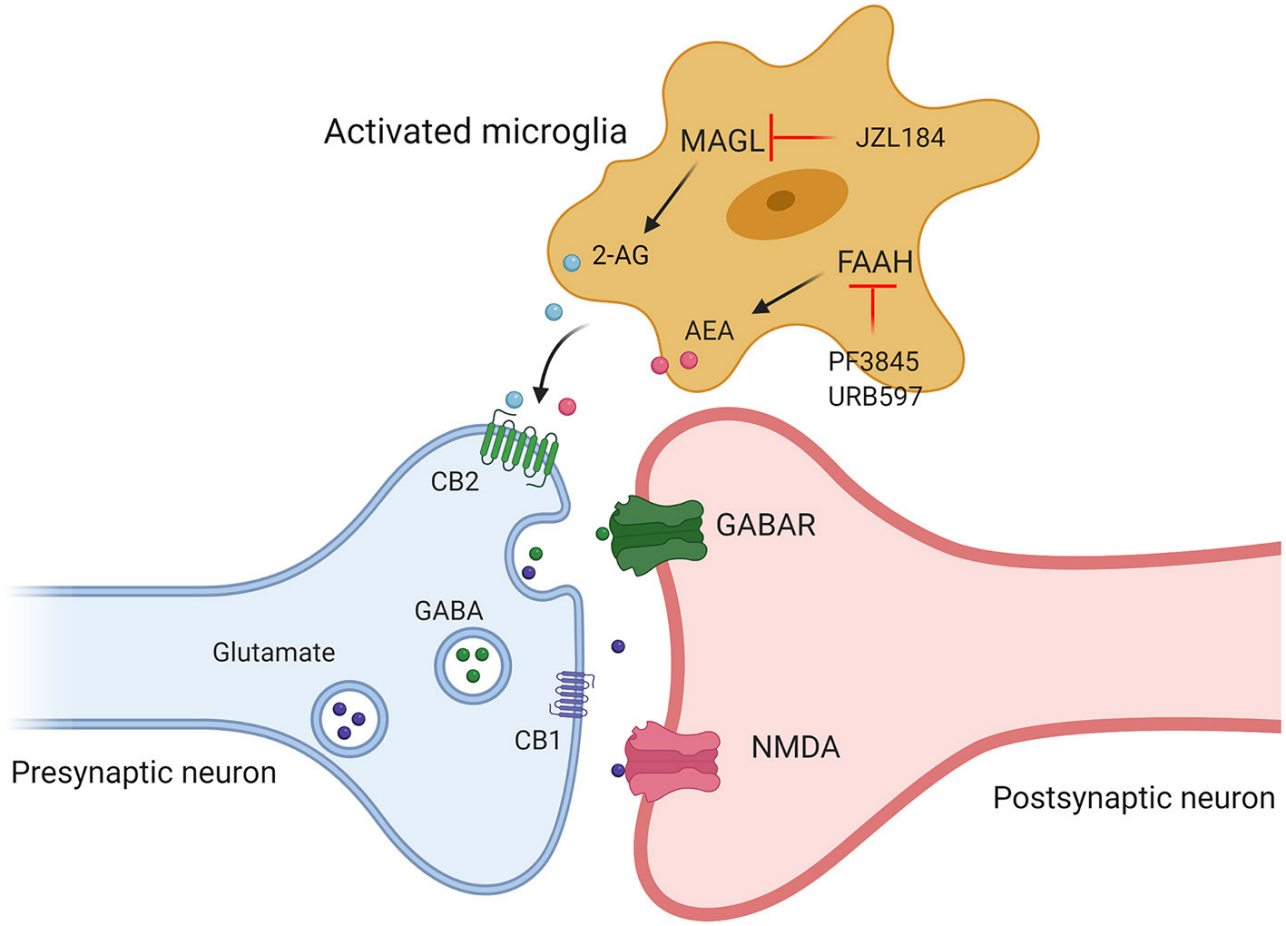
Targeting different components of ECS (2-AG, AEA, CB1, and CB2) may provide therapeutic strategies for the treatment of ASD. Administration of JZL184, a pharmacological inhibitor of MAGL, enhanced 2-AG signaling, by using FAAH inhibitor (PF3845, URB597) to increase AEA levels to promote the release of excitatory and inhibitory amino acid transmitters by acting on cannabinoid receptors.

The above animal and clinical studies provide solid evidence that unbalanced ECS signaling are involved in the occurrence of ASD. Therefore, modulating ECS signaling by targeting different components of ECS (2-AG, AEA, CB1, and CB2) may provide therapeutic strategies for the treatment of ASD (Figure 3). In the last decade, especially in 2018, FDA approved the application of CBD-rich Epidiolex in the treatment of refractory epilepsy, a common comorbid symptom of autism, and CBD and ECS have received extensive attention for the pathophysiological mechanism and therapeutic effect of neurological diseases in children. Currently, most of the studies on the relationship between ASD and ECS are still in the preclinical stage. Many histological and neurochemical abnormalities have been reported in ASD patients and animal models, reflecting the heterogeneity and complexity of this group of diseases, which poses a serious challenge to study on clinical treatment and hinders the identification of possible common pathophysiological mechanisms of ASD. Microglia are indispensable coordinators of CNS development and homeostasis, and possibly participated in the pathogenesis of ASD. ECS signaling can regulate the activity and functions of microglia (82). Both clinical and preclinical studies demonstrate that the neuropathology of ASD is characterized by an abnormal inflammatory status of the brain. Therefore, a detailed investigation of the roles of CB2 receptors and related ligands may shed new light on the pathophysiological mechanisms and therapeutic targets of autism.

## Author Contributions

LP and JY made manuscript planning and structuring. YY and QL did literature searching. TS drafted the manuscript. YY, QL, and JY revised the manuscript. TS and LP edited the final manuscript. All authors have read and approved the final manuscript.

## Funding

This study was supported by grants from the National Natural Science Foundation of China (Nos. 81804188 and 81870932).

## Conflict of Interest

The authors declare that the research was conducted in the absence of any commercial or financial relationships that could be construed as a potential conflict of interest.

## Publisher's Note

All claims expressed in this article are solely those of the authors and do not necessarily represent those of their affiliated organizations, or those of the publisher, the editors and the reviewers. Any product that may be evaluated in this article, or claim that may be made by its manufacturer, is not guaranteed or endorsed by the publisher.
